# Functions that Protect *Escherichia coli* from Tightly Bound DNA-Protein Complexes Created by Mutant EcoRII Methyltransferase

**DOI:** 10.1371/journal.pone.0128092

**Published:** 2015-05-19

**Authors:** Morgan L. Henderson, Kenneth N Kreuzer

**Affiliations:** 1 University Program in Genetics and Genomics, Duke University Medical Center, Durham NC, 27710, United States of America; 2 Department of Biochemistry, Duke University Medical Center, Durham NC, 27710, United States of America; University of Massachusetts, UNITED STATES

## Abstract

Expression of mutant EcoRII methyltransferase protein (M.EcoRII-C186A) in *Escherichia coli* leads to tightly bound DNA-protein complexes (TBCs), located sporadically on the chromosome rather than in tandem arrays. The mechanisms behind the lethality induced by such sporadic TBCs are not well studied, nor is it clear whether very tight binding but non-covalent complexes are processed in the same way as covalent DNA-protein crosslinks (DPCs). Using 2D gel electrophoresis, we found that TBCs induced by M.EcoRII-C186A block replication forks *in vivo*. Specific bubble molecules were detected as spots on the 2D gel, only when M.EcoRII-C186A was induced, and a mutation that eliminates a specific EcoRII methylation site led to disappearance of the corresponding spot. We also performed a candidate gene screen for mutants that are hypersensitive to TBCs induced by M.EcoRII-C186A. We found several gene products necessary for protection against these TBCs that are known to also protect against DPCs induced with wild-type M.EcoRII (after 5-azacytidine incorporation): RecA, RecBC, RecG, RuvABC, UvrD, FtsK, XerCD and SsrA (tmRNA). In contrast, the RecFOR pathway and Rep helicase are needed for protection against TBCs but not DPCs induced by M.EcoRII. We propose that stalled fork processing by RecFOR and RecA promotes release of tightly bound (but non-covalent) blocking proteins, perhaps by licensing Rep helicase-driven dissociation of the blocking M.EcoRII-C186A. Our studies also argued against the involvement of several proteins that might be expected to protect against TBCs. We took the opportunity to directly compare the sensitivity of all tested mutants to two quinolone antibiotics, which target bacterial type II topoisomerases and induce a unique form of DPC. We uncovered *rep*, *ftsK* and *xerCD* as novel quinolone hypersensitive mutants, and also obtained evidence against the involvement of a number of functions that might be expected to protect against quinolones.

## Introduction

When the DNA replication machinery encounters DNA damage of various kinds, or unusual situations such as secondary structures, DNA crosslinks and tightly bound proteins, replication fork blockage can occur (for reviews, see [1,2]). Blocked replication forks in turn can induce DNA damage responses, and can also lead to mutation or cell death. Recent work has begun to unravel multiple pathways that cells use to restore active replication after fork blockage; some of these involve damage repair while others simply avoid the damage, leaving it in one of the daughter duplexes (for reviews, see [3,4,5]).

A particularly difficult and poorly studied form of damage involves DNA-protein crosslinks (DPCs). A number of DNA damaging agents, including ultraviolet light (UV), ionizing radiation, formaldehyde, certain carcinogens, and some chemotherapeutic drugs, cause proteins to become trapped on DNA in DPCs, which have been shown to block DNA replication [6,7,8]. A challenge in studying DPCs is that many inducing agents, such as radiation and formaldehyde, create DPCs throughout the chromosomal DNA and the crosslinks involve a wide variety of different DNA-binding proteins [6,7]. Other inducing agents, such as chemotherapeutic drugs that target DNA topoisomerases and DNA methyltransferases, lead to DPCs that are both DNA site-specific and protein-specific. While drug-induced DPCs involving topoisomerases are complex (contain hidden DNA breaks) and generally reversible, drug-induced DPCs with DNA methyltransferases contain a simple DNA-protein crosslink at the active site of the protein and are very long-lived or non-reversible. For example, 5-azacytidine (aza-C), a cytidine analog with nitrogen at the C5 position, traps various cytosine methyltransferases including EcoRII methyltransferase (M.EcoRII) in DPCs at the cognate recognition site of the enzyme in DNA [9,10]. For these reasons, several groups have used aza-C-induced M.EcoRII DPCs as a model system to investigate how cells respond to and repair DPCs.

Replication can also be blocked by tightly bound but non-covalent complexes [11,12] (also see below), and it is not clear whether the processing and consequences of tightly bound DNA-protein complexes (TBCs) are similar to those of DPCs. One system that has been used to study the consequences of replication fork blocks created by TBCs involves tandem repressor-operator complexes. *E*. *coli* strains expressing either TetR or LacI repressor, with tandem arrays of 240 copies of the respective binding sites (*tetO* or *lacO*) in the chromosome, demonstrated site-specific and reversible blockage of chromosomal replication at the TBC array [12]. Replication sometimes proceeded some distance into the array, suggesting limited success at replication through the blockage. When the TBCs were present for 2 hours and then reversed by addition of the appropriate inducer, replication was shown to rapidly restart in either wild-type or *recA* mutant cells [12]. However, maintaining the chromosomal TBC array for 4 hours in wild-type cells led to a massive loss of viability, and so eventually some toxic event is induced by the long TBC arrays [12].

Shorter TBC arrays (22 or 34 copies) were also found to create replication fork blockage *in vivo*, and arrays even shorter than that blocked replication *in vitro* but not *in vivo* [13]. These results suggested that factors, such as recombination proteins and/or helicases, can prevent or ameliorate fork blockage *in vivo*. Interestingly, RecA, RecBCD, and RecG were all required for cell viability in the presence of the 34-copy TBC arrays, whereas RecF, Rep, UvrD, and RuvABC were not [13]. These genetic results imply that double-strand breaks (DSBs) are somehow formed in response to the replication blocks. While *rep* and *uvrD* single mutants were viable with the 34-copy TBC arrays, a later study revealed a synthetic phenotype indicating redundant roles of Rep and UvrD in promoting replication through the 34-copy TBC array [14].

Tandem arrays of TBCs present a rather unnatural obstacle to DNA replication, and their processing and consequences might be distinct from single TBCs. Two different tight binding proteins, active site (catalytically inactive) mutants of a restriction nuclease and a restriction methyltransferase, have been used to generate solo, sporadically located (non-arrayed), TBCs. The E111G mutant of EcoRI nuclease binds very tightly to its recognition sequence and causes lethality when expressed at high levels [15], as does the C186A mutant of M.EcoRII [16,17,18]. The E111G EcoRI protein blocks *E*. *coli* replication forks *in vitro*, and either Rep or UvrD helicase (but not RecG, PriA or Mfd) can relieve the replication block [14]. TBCs created with a mutant EcoRI nuclease were also shown to block transcription *in vitro* [19], although a later study showed that additional trailing RNA polymerases can allow the transcription complex to push through the TBC, dislodging the protein in the process [20].

The above studies show that solo TBCs are potent replication and transcription blocks *in vitro*, and that they can be cytotoxic *in vivo*. However, they do not illuminate the *in vivo* molecular consequences nor the pathways that cells might use to overcome solo TBCs. The studies with both tandem and solo TBCs also support redundant roles for Rep and UvrD in overcoming protein blockage of various types. Several studies argue that the most important and/or common fork blockage *in vivo* is caused by collisions with RNA polymerase [14,21], and it is possible (though untested) that TBCs such as mutant restriction system enzymes mimic the effect of RNA polymerase with regard to replication fork blockage. As will be discussed below, recent studies provide evidence that Rep plays a special role at blocked replication forks while UvrD plays a special role at blocked transcription complexes (see Further Discussion) [14,22,23,24].

It is important to note that bacterial cells maintain a specialized and intentional fork-blockage system to assist in the completion of DNA replication. *E*. *coli* replication forks are paused, in a unidirectional fashion, when the forks encounter the Tus protein bound to *Ter* DNA sites in the terminus region [25,26,27]. When *Ter* sites are inserted in regions outside the terminus, RecBCD-mediated homologous recombination (HR) and induction of the SOS response are required to tolerate the blocked replication forks [28,29,30]. UvrD is also required for survival in this situation, which was traced to the ability of UvrD to remove the bound Tus from *Ter* sites [31,32].

Two other fork blockage systems have been studied in some detail and provide an interesting comparison to the systems described above. Following UV exposure, the replication machinery can be blocked by pyrimidine dimers [33,34]. RecA, along with loading factors RecF, RecO and RecR, are needed to stabilize the arrested replication fork from extensive degradation after UV damage, while RecJ exonuclease and RecQ helicase degrade nascent lagging strand DNA at the blocked fork [35,36,37,38,39]. Furthermore, RecA is required for the processing of these blocked forks [40], and *ruvAB* and *ruvC* mutants accumulate unresolved Holliday junctions after UV treatment [41]. Rep helicase, along with primosome proteins PriB and PriA, are required for restarting replication in this system [42]. The second system involves fork blockage after temperature shift of a DnaB^ts^ mutant, which can lead to the formation of RuvABC-mediated DSBs [43]. In this case, the presence of RecA contributes to DSB formation, presumably by RecA-mediated Holliday junction formation for RuvABC action [44]. The forks stalled following DnaB inactivation are degraded by RecFOR, along with RecJ, RecG, and ExoI (SbcB) [45].

In this study, we analyze the *in vivo* consequences and protein requirements for survival upon expression of the tight-binding mutant M.EcoRII protein (C186A), which creates solo TBCs. Using 2D gel electrophoresis, we visualized that a single TBC can block replication forks *in vivo*, as was previously shown for covalent DPCs [8]. Interestingly, based on the molecular forms observed, the downstream consequences of fork blockage are different depending on whether M.EcoRII is in a covalent or non-covalent complex on the DNA. We also performed an extensive candidate gene screen for mutants that are hypersensitive to the M.EcoRII-induced solo TBCs, using an arabinose expression system that allows carefully titrated expression of M.EcoRII. A powerful aspect of this system is that previous studies, from our lab and others, have already identified a variety of mutants that are hypersensitive to aza-C-induced DPCs that involve the same M.EcoRII protein (except that the DPCs utilize the wild-type version of the protein) ([8,46,47,48,49,50,51,52]; reviewed in [7]). We also took the opportunity to test the sensitivity of each mutant to two of the quinolone antibiotics, which target the bacterial type II topoisomerases [53,54,55]. Quinolones stabilize a unique form of DPC, called the cleavage complex, in which topoisomerase is covalently bound to the two ends of a staggered double-strand DNA break. Like DPCs, quinolone-stabilized DNA gyrase cleavage complexes block replication forks *in vivo* [56]. The mechanism(s) by which quinolone-stabilized cleavage complexes lead to DSB formation and ultimately cell death have been extensively studied but are still not well understood at the molecular level (for reviews, see [57,58,59,60]). Overall, our results support a model in which RecFOR/RecA-dependent fork processing promotes dissociation of a TBC, and also reveal that the tmRNA system helps protect against TBCs, presumably helping to overcome TBC-mediated blockage of coupled transcription-translation complexes. Interestingly, different but overlapping functions are involved in surviving TBCs versus DPCs, both involving the M.EcoRII protein.

## Materials and Methods

### Materials

Restriction enzymes were from New England Biolabs; Nytran membranes from Schleicher & Schuell; QuickChange Mutagenesis Kit from Stratagene; Random Primed DNA Labeling Kit from Roche Applied Science; and radiolabeled nucleotides from Perkin-Elmer Life Science. LB (Luria broth) contained bacto tryptone (10 g/L), yeast extract (5 g/L), and sodium chloride (10 g/L).

### Plasmids

Plasmid pBAD-MEcoRII contains the wild-type M.EcoRII coding sequence between the KpnI and SphI sites in the multiple cloning region of pBad33, downstream of the *araBAD* promoter [51]. Plasmid pBAD-MEcoRII contains a chloramphenicol resistance gene and is based on the pACYC184 replicon. Plasmid pBAD-MEcoRII-C186A, containing the active site mutant version of the M.EcoRII coding sequence, was created by mutating the active site cysteine codon of M.EcoRII within plasmid pBAD-MEcoRII using Stratagene QuickChange Mutagenesis Kit [17]. Plasmid pBR322-C1060A is a derivative of pBR322 with a cytosine to adenine mutation at location 1060, destroying one M.EcoRII recognition site in the plasmid [8].

### *E*. *coli* strains

Strain BW27783 [F-, *Δ(araD-araB)567*, *ΔlacZ4787(*::*rrnB-3)*, λ-, *Δ(araH-araF)570(*::*FRT)*, *ΔaraEp-532*::*FRT*, φ*Pcp8araE535*, *rph-1*, *Δ(rhaD-rhaB)568*, *hsdR514*)] allows for homogenous and titratable expression from pBAD vectors [61] and was obtained from the lab of HP Erickson (Duke University). *E*. *coli* knockout mutants from the Keio collection [62] contain kanamycin resistance gene inserts, and are identified in this paper as *∆* followed by gene name (e.g. *∆recA*). *E*. *coli* transposon mutants (kanamycin resistance gene) were created in a recent genetic screen [51,52] and are identified here as insertions (e.g. *ssrA*::*Kan*). In each case, the desired mutation was moved into the BW27783 background via P1 transduction [63], selecting for kanamycin resistance, and confirmed by PCR. The kanamycin-resistance cassette was not removed from the tested strains. The *recF4115* missense mutant (K36Q) was moved into BW27783 using P1 transduction, selecting for a linked Tn10 marker (in *tnaA;* [64]); transductants were screened by DNA sequencing for co-transduction of the *recF* mutation.

### Spot tests for sensitivity to M.EcoRII C186A and quinolone antibiotics

Overnight cultures of BW27783 (wild-type) and the indicated BW27783 derivatives, containing plasmid pBAD-MEcoRII-C186A, were diluted to the equivalent of OD_560_ = 5x10^-4^ (corresponding to approximately 2 x 10^5^ cells per mL). Five-fold serial dilutions were generated across a microtiter plate and 5 μl of each dilution (range of roughly 1,000–0.3 colony forming units for wild-type) was spotted onto LB plates containing chloramphenicol with either glucose (0.01%) or arabinose (0.0003%, 0.001%, 0.003%, or 0.01%). The same dilutions were also spotted onto LB plates containing either ciprofloxicin (5 ng/mL or 7.5 ng/mL) or nalidixic acid (1.5 μg/mL or 2.5 μg/mL). All plates were incubated at 37° overnight. Spot tests were performed on each strain at least three times, and representative examples are shown.

### 2D gels to visualize DNA intermediates

Replication intermediate were visualized by 2D gel electrophoresis using a procedure similar to that of Kuo et al [8]. Overnight cultures of BW27783 containing plasmid pBAD-MEcoRII-C186A and either pBR322 or pBR322-C1060A were grown with and without 0.01% glucose until OD_560_ = 0.3. Arabinose (0.00005%) was then added to the no-glucose culture and 4-mL samples were collected after 60 min at 37°C. Samples were pelleted and frozen at -80°C. Cell pellets were resuspended in 500 μL of Triton lysis butter [50 mM Tris-HCl (pH 7.8), 10 mM EDTA, 1% Triton X-100, and lysozyme at 1.8 mg/mL] and incubated at 65°C for 20 min. Proteinase K (0.5 mg/mL) and SDS (0.2%) were added to the samples and incubated at 55°C for 1 h. DNA was extracted with phenol/chloroform/isoamyl alcohol (25:24:1) and dialyzed against TE [10 mM Tris-HCl (pH 7.8), 1 mM EDTA] at 4°C overnight. The DNA (50 μL) was digested with Pst1-HF for 2 h at 37°C. Digested DNA was separated by size in the first dimension gel (0.4% agarose) run in 0.5X Tris-borate EDTA (TBE) buffer at 1 V/cm for 29 hours. The desired slices were cut from the gel, rotated 90° counterclockwise, and cast within the top of the second-dimension gel (1% agarose with ethidium bromide at 0.3 ug/mL). The second dimension gel was run at 4.5 V/cm for 15 hours at 4°C with recirculated 0.5X TBE containing ethidium bromide (0.3 μg/mL). The gels were analyzed by Southern hybridization with a 1,158-bp gene probe from pBR322 that does not hybridize with plasmid pBAD-MEcoRII-C186A. The probe was generated by PCR amplification from plasmid pBR322 using primers 5’-CGGTATTCGGAATCTTGCAC-3’ and 5’-AGCTCGTTGAGTTTCTCCAG-3’ and purified using the DNA Clean & Concentrator Kit (Genesee Scientific). The probe was ^32^P-labeled using the Random Primed DNA Labeling Kit (Roche Applied Science). Southern blots were visualized by PhosphorImager.

## Results and Discussion

### Fork blockage at TBCs created by M.EcoRII-C186A

**Fig 1:**
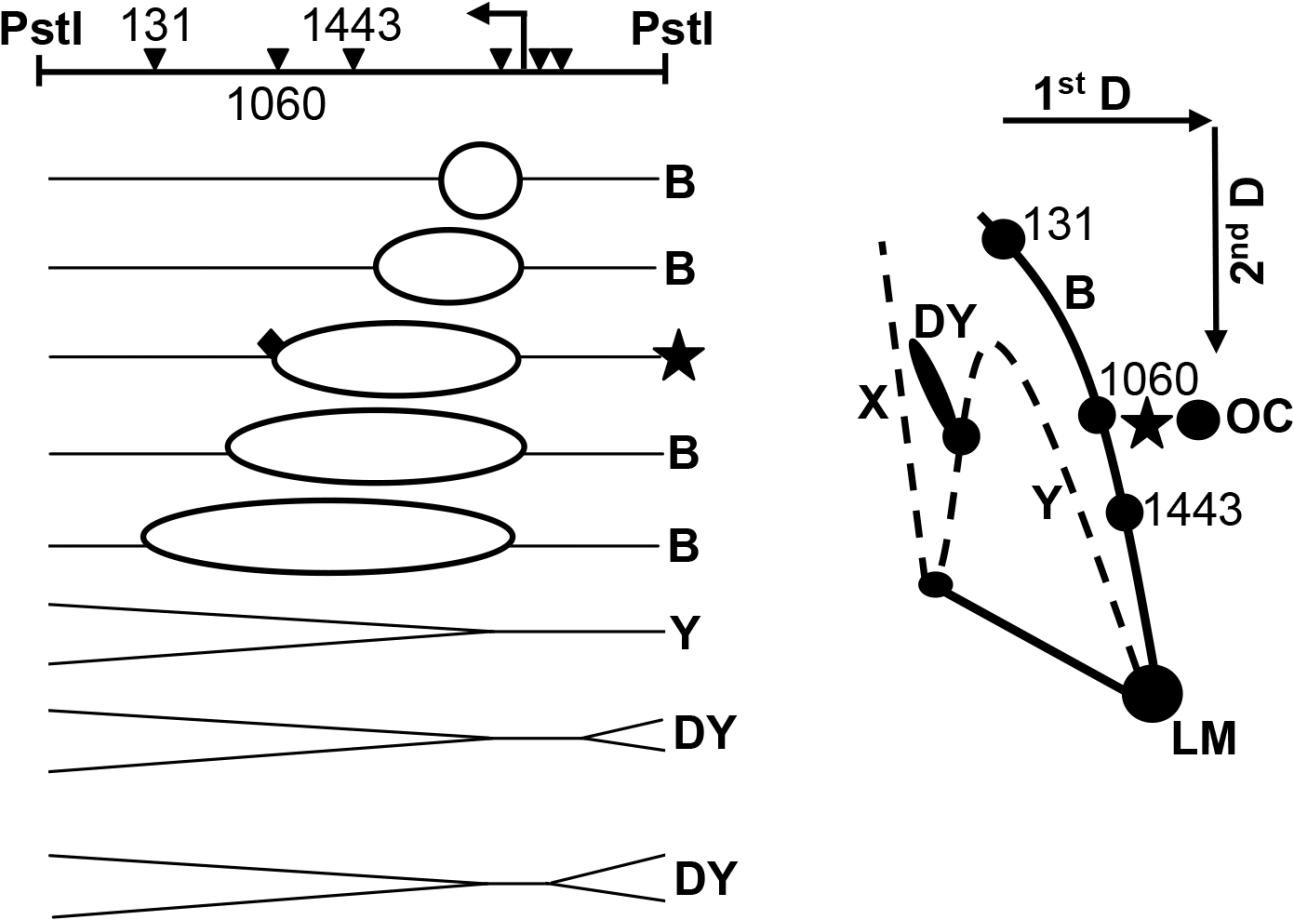
Predicted replication intermediates and two-dimensional gel pattern of linearized pBR322. The diagram on the left depicts plasmid pBR322 linearized by PstI at 3,607 bp; *leftward arrows*, replication start site (2,535 bp). *Inverted triangles*, *Eco*RII methylation sites (at positions 131, 1,060, 1,443, 2,501, 2,622, and 2,635 bp from left to right); *diamond*, tightly bound MTase at a blocked fork. The diagram on the right depicts *B*, bubble; *DY*, double *Y*; X, X structures; *OC*, open circle; *LM*, linear monomer; *star*, replication intermediate blocked at the 1,060 methylation site and the resulting accumulation on the two-dimensional gel.

**Fig 2:**
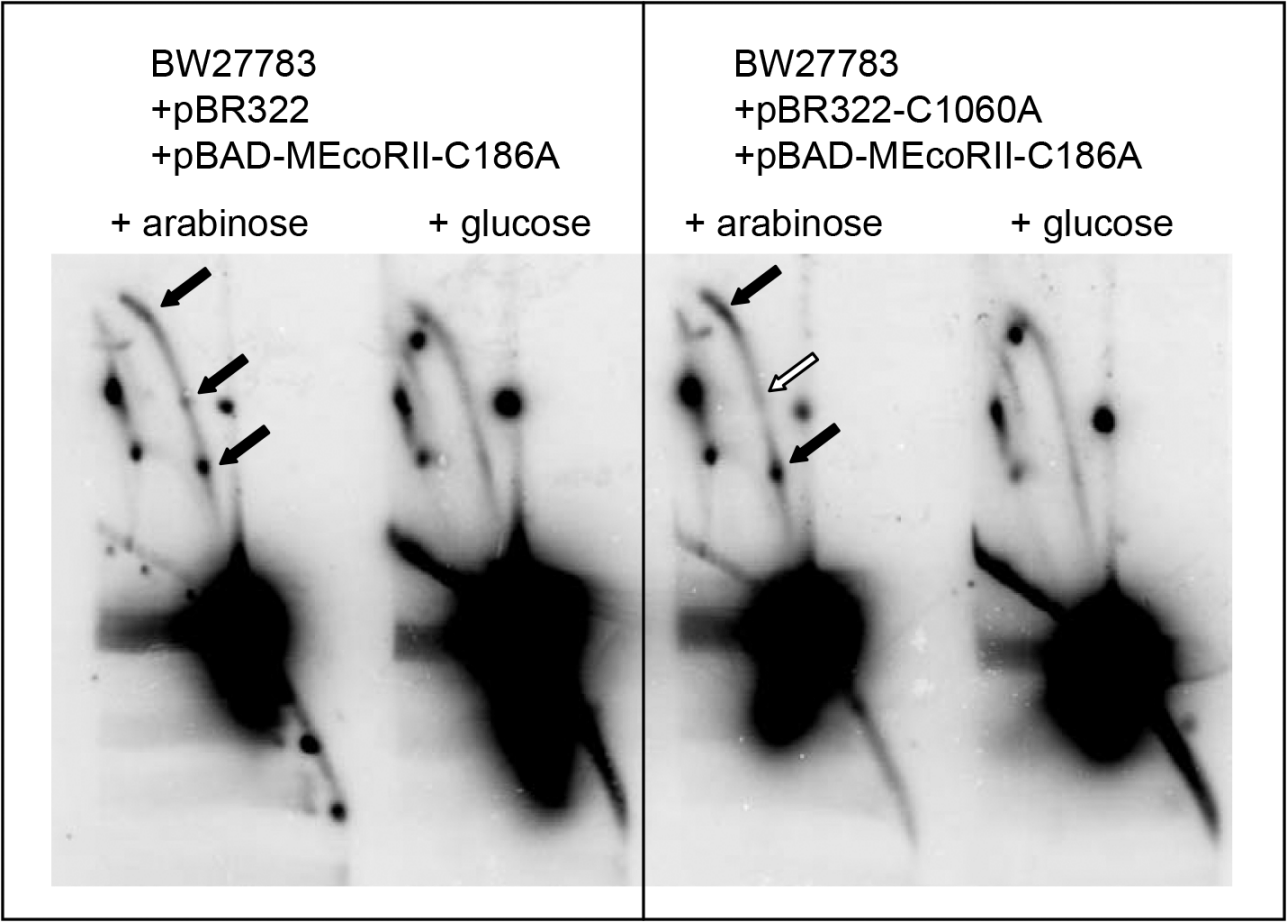
DNA replication is blocked in cells expressing mutant *Eco*RII C186A methyltransferase. DNA was digested with Pst1-HF, run on two-dimensional gel electrophoresis and visualized with Southern hybridization. *Closed arrows* show accumulation of bubble molecules at locations consistent with the *Eco*RII methylation sites at 131, 1,060, and 1443 bp. *Open arrow* shows disappearance of the 1,060 bp spot with the mutated pBR322-C1060A plasmid.

Previous work from our lab used 2D agarose gel electrophoresis to visualize the accumulation of replication forks blocked at aza-C-induced DPCs involving the wild-type M.EcoRII protein [8]. We used the same 2D gel electrophoresis technique (Fig 1) to determine whether a single tightly bound (but non-covalent) M.EcoRII complex could also block replication forks. We found an accumulation of bubble molecules at locations consistent with the EcoRII methylation sites at 1,443 and 1,060 bp (and less convincingly at 131 bp) (Fig 2). The spot corresponding to the 1,443-bp recognition site was consistently stronger than the other two, suggesting that M.EcoRII sites may be saturated with the mutant M.EcoRII protein, leading to more frequent fork blockage at sites that are encountered earlier in replication of the plasmid (i.e. close to the replication origin; note that the M.EcoRII site at position 2,501 is very close to the origin—we do not know whether forks are blocked at that site, since the resulting bubble spot would be difficult or impossible to detect in the vicinity of the very intense monomer spot). The spots on the bubble arc were only detected with the addition of arabinose to induce expression of M.EcoRII-C186A.

To directly test whether TBC formation at M.EcoRII sites was leading to the blocked replication forks, we analyzed a mutated pBR322-C1060A plasmid, which lacks the methylation site at 1,060 bp. Indeed, the bubble spot corresponding to the 1,060-bp site disappeared while the spot corresponding to the 1,443-bp site remained, confirming that the accumulation of bubble molecules is due to blockage at *Eco*RII methylation sites. We conclude that solo TBCs involving the mutant M.EcoRII protein are capable of blocking replication forks *in vivo*, as do DPCs formed after aza-C treatment with wild-type M.EcoRII.

Strikingly, the 2D gel patterns after aza-C-induced DPC formation with wild-type M.EcoRII [8] showed two prominent features that were not seen here after TBC formation with M.EcoRII-C186A. First, aza-C-induced DPCs caused an accumulation of RecA-dependent X structures, presumably due to RecA-dependent recombination or replication fork regression. Second, a prominent Y-arc with spots was seen after DPC formation, and attributed to the induction of rolling-circle replication (confirmed by EM analysis). These results indicate that DPCs lead to more frequent fork processing events than TBCs (see Further Discussion).

### Functions that protect from TBCs created by M.EcoRII-C186A

To determine which proteins play a role in protecting from the damage created by TBCs induced by M.EcoRII-C186A, a collection of knockout mutants was created in a genetic background that allows carefully titrated expression from an arabinose promoter. We then compared sensitivities by examining growth (or lack thereof) in the presence of increasing concentrations of arabinose. The collection was largely chosen based on known roles in protection from DPCs or other damage that leads to stalled replication forks. The proteins could be involved in directly reversing the TBC, protecting from the immediate consequences of the TBC (e.g. restarting replication forks or dealing with stalled transcription/translation), or repairing downstream damage from the TBCs (e.g. broken replication forks).

### Recombination proteins and sensitivity to TBCs

**Fig 3:**
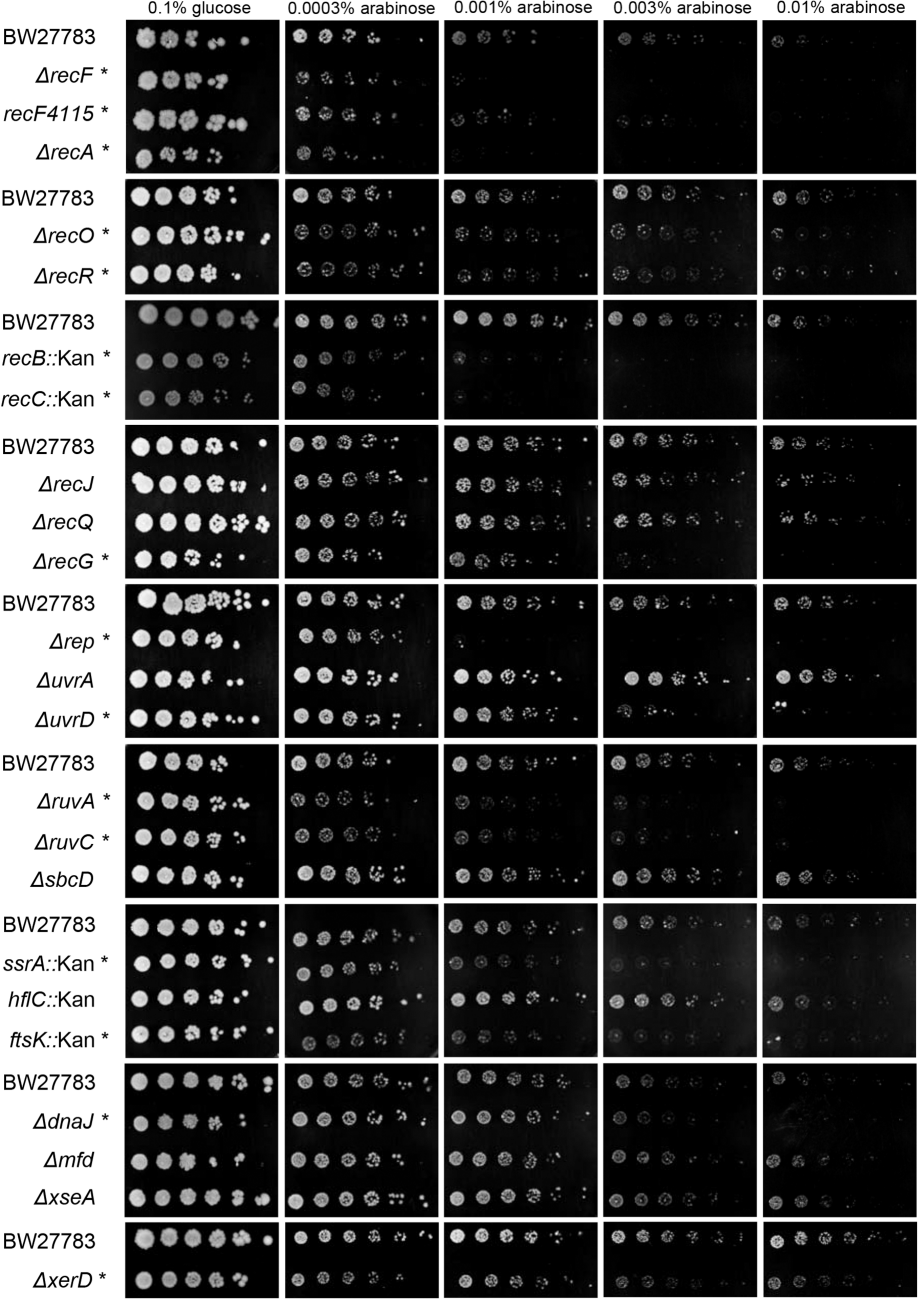
Sensitivity to M.EcoRII C186A. Overnight cultures of BW27783 (wild-type) and the indicated BW27783 derivatives, containing plasmid pBAD-MEcoRII-C186A, were serial diluted five-fold and spotted onto LB plates containing chloramphenicol with either glucose (0.01%) or arabinose (0.0003%, 0.001%, 0.003%, or 0.01%) and incubated at 37° overnight. Those mutants deemed to be hypersensitive are indicated with an asterisk.

Recombination and repair proteins play important roles in survival from damage due to DPCs and topoisomerase cleavage complexes. For example, RecA, which catalyzes strand exchange, and RecBCD, which prepares broken ends for HR, greatly improve survival from aza-C-induced DPCs [8,46,47,49,52,65] and quinolone-induced cleavage complexes [66,67]. We found that *recA*, *recB* and *recC* mutants were all markedly sensitive to TBCs generated by expression of M.EcoRII-C186A (Fig 3; data summarized in Table 1). Therefore, DSBs are apparently generated after formation of both DPCs and TBCs. RecA and RecBCD may play a direct role in repairing DNA-protein complexes (presumably with DSB formation), or alternatively, these proteins may be involved in repairing downstream DNA damage such as replication forks that are broken after fork blockage.

**Table 1:** Summary of growth in the presence of arabinose-induced TBCs.

Mutant	Glucose	0.0003% Ara	0.001% Ara	0.003% Ara	0.01% Ara
*ΔrecF* *	0	+1 ↓	-3 ↓	-4 ↓	-2 ↓
*recF4115* *	+1	0 ↓	0 ↓	-1 ↓	-2 ↓
*ΔrecA* *	0	0 ↓	-2 ↓	-4 ↓	-2 ↓
*ΔrecO* *	+1	+1 ↓	0 ↓	-1 ↓	-4 ↓
*ΔrecR* *	0	0 ↓	+1 ↓	0 ↓	-4 ↓
*recB*::*Kan* *	-1 ↓	0 ↓	-5 ↓	-6 ↓	-4 ↓
*recC*::*Kan* *	-1 ↓	-1 ↓	-5 ↓	-6 ↓	-4 ↓
*ΔrecJ*	+1	0	0	0	0
*ΔrecQ*	+2	0	0	+1	0
*ΔrecG* *	0	-1	0 ↓	-2 ↓	-4 ↓
*Δrep* *	-1	0	-5 ↓	-5 ↓	-4 ↓
*ΔuvrA*	0	0	0	0	0
*ΔuvrD* *	0	0	-1	-2 ↓	-3 ↓
*ΔruvA* *	+1	0 ↓	-1 ↓	-4 ↓	-5 ↓
*ΔruvC* *	+1	0 ↓	-1 ↓	-3 ↓	-4 ↓
*ΔsbcD*	+1	+1	+1	+1	0
*ssrA*::*Kan* *	0	0	-1 ↓	-4 ↓	-5 ↓
*hflC*::*Kan*	0	-1	0	-1	0
*ftsK*:*Kan* *	0	0 ↓	-2 ↓	-2 ↓	-4 ↓
*ΔdnaJ* *	-1	0	+1	-1	-3 ↓
*Δmfd*	-1	-1	0	0	+1
*ΔxseA*	0	0	+1	0	+1
*ΔxerD* *	0	-1 ↓	0 ↓	-1 ↓	-2 ↓

Results of dilution plating (Fig 3) were categorized in comparison to wild-type dilution series on the same plate. The number in each entry indicates which dilution tube showed growth (more than one colony) relative to the wild-type series (+1 indicates that the mutant grew in the next more dilute spot than the wild type while -1 indicates that the mutant grew in the next more concentrated spot; note that each dilution step was five-fold, and so a reading of -3 would correspond to a dilution factor differential of 125). The differential in 0.01% arabinose (Ara) is sometimes not as dramatic as that in 0.003% arabinose because the wild type did not grow in the more dilute spots on the 0.01% plate (not because the mutant grew better at the higher arabinose concentration). The downward arrow next to the number indicates that the colonies that did grow were noticeably smaller than the corresponding wild-type colonies on the same plate or that no colonies grew in the corresponding mutant series. Both negative numbers and downward arrows provide evidence for hypersensitivity.

* Mutants designated as hypersensitive are indicated by an asterisk.

Like RecBCD, RecFOR assists in RecA-mediated recombination, but RecFOR proteins target RecA to single-stranded gaps rather than DNA ends ([36,68]; also see Introduction). We found that *recF*, *recO* and *recR* mutants are hypersensitive to TBCs induced by M.*Eco*RII-C186A (Fig 3; Table 1). Notably, *recFOR* mutants are not sensitive to aza-C-induced DPCs [48,52], nor are they sensitive to quinolones ([67]; also see Further Discussion below). Mutants without RecJ or RecQ showed little or no sensitivity to M.EcoRII-C186A-induced TBCs, indicating that erosion of the nascent lagging strand is not relevant to the processing pathway (Fig 3; Table 1; the *recJ* and *recQ* mutants sometimes appeared to be very slightly sensitive, but not in a convincing and reproducible manner).

RuvA and RuvC are subunits of the Resolvasome, which resolves Holliday junctions and contributes to rescue of blocked DNA replication forks through replication fork reversal [43,69]. RuvA and RuvC knockout mutants are hypersensitive to aza-C-induced DPCs [49,52] and to quinolone-induced cleavage complexes [67,70]. We found that *ruvA* and *ruvC* mutants are also hypersensitive to TBCs induced by M.EcoRII-C186A (Fig 3; Table 1). Together with the evidence for TBC-induced fork blockage and hypersensitivity of *recBC* mutants (see above), we speculate that TBC-blocked replication forks are sometimes cleaved by a nuclease to generate broken forks, which need to be repaired by RecBCD- and RuvABC-dependent HR. In this view, the hypersensitivity of *ruvA* and *ruvC* mutants suggests that some other nuclease is responsible for fork cleavage. An equally plausible explanation is that breaks are generated when a second replication fork collides with the stalled fork from behind, as seen with ectopically located Ter sites in the *E*. *coli* chromosome [30].

One candidate for a nuclease that might cleave blocked forks, or even DNA near the tightly bound protein, is SbcCD. This protein has double-strand DNA exonuclease activity as well as single-strand DNA endonuclease activity [71], and has been shown to cleave palindromic branched DNA [72] as well as DNA near a tightly bound protein (streptavidin bound at biotin-tagged DNA end) [73]. We found that *sbcD* mutants are not hypersensitive to TBCs induced by M.EcoRII-C186A (Fig 3; Table 1). Knockouts of SbcCD also display wild-type sensitivity to aza-C-induced DPCs [52].

### Helicases and sensitivity to TBCs

Helicases function in diverse cellular processes, and multiple studies implicate various helicases in responding to DNA-bound proteins. For example, Rep helicase allows replication fork progression past a protein-DNA complex [14,74,75], and also prevents the formation of DSBs following replication fork arrest [76]. UvrD helicase functions in nucleotide excision repair (NER) and mismatch repair (MMR) and can remove the replication terminator protein, Tus, bound at *Ter* sites or RecA protein from filaments [31,32,77]. We found that strains lacking either Rep or UvrD helicase are hypersensitive to TBCs induced by M.EcoRII-C186A, with the *rep* mutant being the more hypersensitive of the two (Fig 3; Table 1).

Notably, the response to aza-C induced DPCs involving M.EcoRII was distinct, with *uvrD* mutants being hypersensitive, but not *rep* mutants [49,52]. Given Rep’s well-studied ability to remove proteins bound to DNA, it seems likely that Rep removes the mutant M.EcoRII from TBCs but is unable to remove M.EcoRII from aza-C-induced DPCs, due to the covalent linkage (see Further Discussion). A knockout of UvrA, which functions in nucleotide excision repair, is not sensitive to TBCs induced by M.EcoRII-C186A (Fig 3; Table 1), demonstrating that some UvrD function outside of NER is important. Likewise, a UvrA knockout is not hypersensitive to aza-C-induced DPCs, excluding NER as an important component of DPC repair with these lesions [46,47,49,52,65].

RecG helicase functions in DSB repair and can catalyze branch migration of forked DNA structures and Holliday junctions [40,78]. RecG mutants are hypersensitive to both M.EcoRII-C186A-induced TBCs (Fig 3; Table 1) and aza-C-induced DPCs [49,52]. The role of RecG could involve branch migration during RecBCD-mediated DSB repair and/or modulation of blocked replication forks.

HelD is a helicase that functions in the RecF pathway of HR [79]. Even though the RecF pathway appears to be important in surviving TBCs induced by M.EcoRII-C186A, a *helD* knockout was not hypersensitive (S1 Fig). Finally, DinG helicase has a poorly understood role in DNA repair and replication [80]. We found that a *dinG* knockout is not hypersensitive to TBCs induced by M.EcoRII-C186A (S1 Fig), but we recently showed that it is hypersensitive to aza-C induced DPCs [52], consistent with a special role in repairing DNA-protein crosslinks.

Mfd, the transcription-coupled repair factor in bacteria, removes RNA polymerase stalled at DNA damage, such as UV-induced pyrimidine dimers, and recruits the nucleotide excision repair machinery [81]. Mfd is unable to promote fork movement through the TBC generated by a tight-binding restriction nuclease mutant (EcoRI-E111G) *in vitro* [14], but it does somehow promote the rapid recovery of gene expression following UV-induced DNA damage [82]. An Mfd knockout mutant is not hypersensitive to TBCs induced by M.EcoRII-C186A (Fig 3; Table 1). We previously found that inactivation of Mfd also does not cause hypersensitivity to aza-C-induced DPCs, arguing that it plays no unique role in releasing RNA polymerase stalled at DPCs [49,51].

### Chromosome dimer resolution and the tmRNA pathway protect against TBCs

We tested a number of other knockout mutants, involved in diverse cellular functions that could conceivably play a role in protecting cells from TBCs.

FtsK is an essential cell division protein linking cell division and chromosome segregation, and also stimulates XerCD-mediated chromosome dimer resolution. The N-terminal 210 residues of FtsK is sufficient for the essential cell division functions of the protein, but truncated proteins with this essential region are unable to stimulate XerCD recombination [83]. We previously isolated *ftsK* insertion mutants in a transposon mutagenesis screen for mutants hypersensitive to aza-C-induced DPCs [52]. The transposon in each insertion is located downstream of the coding region for the essential N-terminal segment, suggesting that the relevant defect in these mutants involves XerCD-mediated chromosome dimer resolution. Here, we find that an *ftsK* insertion mutant is also hypersensitive to TBCs induced by M.EcoRII-C186A (Fig 3; Table 1). To test the prediction that this hypersensitivity is caused by a defect in XerCD-mediated recombination, we also tested a *xerD* knockout, which likewise turned out to be hypersensitive to TBCs (Fig 3; Table 1). We conclude that XerCD-mediated chromosome dimer resolution serves to protect *E*. *coli* from detrimental effects of TBCs. This implies that TBCs lead to significant levels of homologous recombination between sister chromosomes, presumably as a result of broken replication forks inferred above (based on sensitivity of *recBC* knockouts).

SsrA (tmRNA) releases stalled ribosomes from the end of an mRNA lacking a stop codon. We showed previously that *ssrA* mutants are hypersensitive to aza-C-induced DPCs, arguing that tmRNA plays an important role in clearing stalled ribosome-mRNA complexes generated after transcription is blocked by aza-C induced DPCs [51]. Likewise, we find that an *ssrA* knockout mutant is hypersensitive to TBCs induced by M.EcoRII-C186A (Fig 3; Table 1). We therefore propose that M.EcoRII-C186A, bound to its recognition sites, can block RNA polymerase and the coupled translation machinery in much the same way as covalently attached M.EcoRII.

Both *dnaJ* and *hflC* mutants are hypersensitive to aza-C-induced DPCs [51]. Either or both of these proteins could potentially function in processing the protein within DPCs, since DnaJ is a chaperone that assists in protein folding, while HflC is part of the HflK-HflC complex which interacts with FtsH to regulate the degradation of various proteins [84,85]. We found that the *hflC* knockout mutant is not hypersensitive to M.EcoRII-C186A, while the dnaJ mutant showed some sensitivity, but only at the highest level of arabinose (Fig 3; Table 1; S1 Fig). Perhaps HflC plays a special role in degrading covalently bound proteins that cannot be dislodged by a helicase (see Further Discussion).

XseA is the large subunit of Exonuclease VII (ExoVII) [86], and ExoVII-deficient mutants are sensitive to both UV irradiation [87] and nalidixic acid [88], for unknown reasons. We found that an *xseA* mutant shows wild-type levels of sensitivity to the TBCs induced by M.EcoRII-C186A (Fig 3; Table 1). Finally, DNA polymerase II (Pol II; encoded by *polB*), functions as a polymerase and exonuclease with reported roles in replication restart following UV exposure, translesion synthesis and nucleotide excision repair (NER) [89,90,91,92,93]. Knockout mutants in *polB* are not hypersensitive to either TBCs induced by M.EcoRII-C186A (S1 Fig) or aza-C induced DPCs [49].

### Proteins that protect from killing by quinolone antibiotics

Quinolone antibiotics stabilize the cleavage complex intermediate of DNA gyrase covalently bound to cleaved DNA, a distinct and pharmacologically important form of DPC. We took the opportunity to test the quinolone (nalidixic acid and ciprofloxacin) sensitivity of all the mutants in this study. This provides comparative data on survival to the various DNA-protein complexes in a common genetic background, extending multiple previous studies of quinolone sensitivities [66,67,70,94].

**Fig 4:**
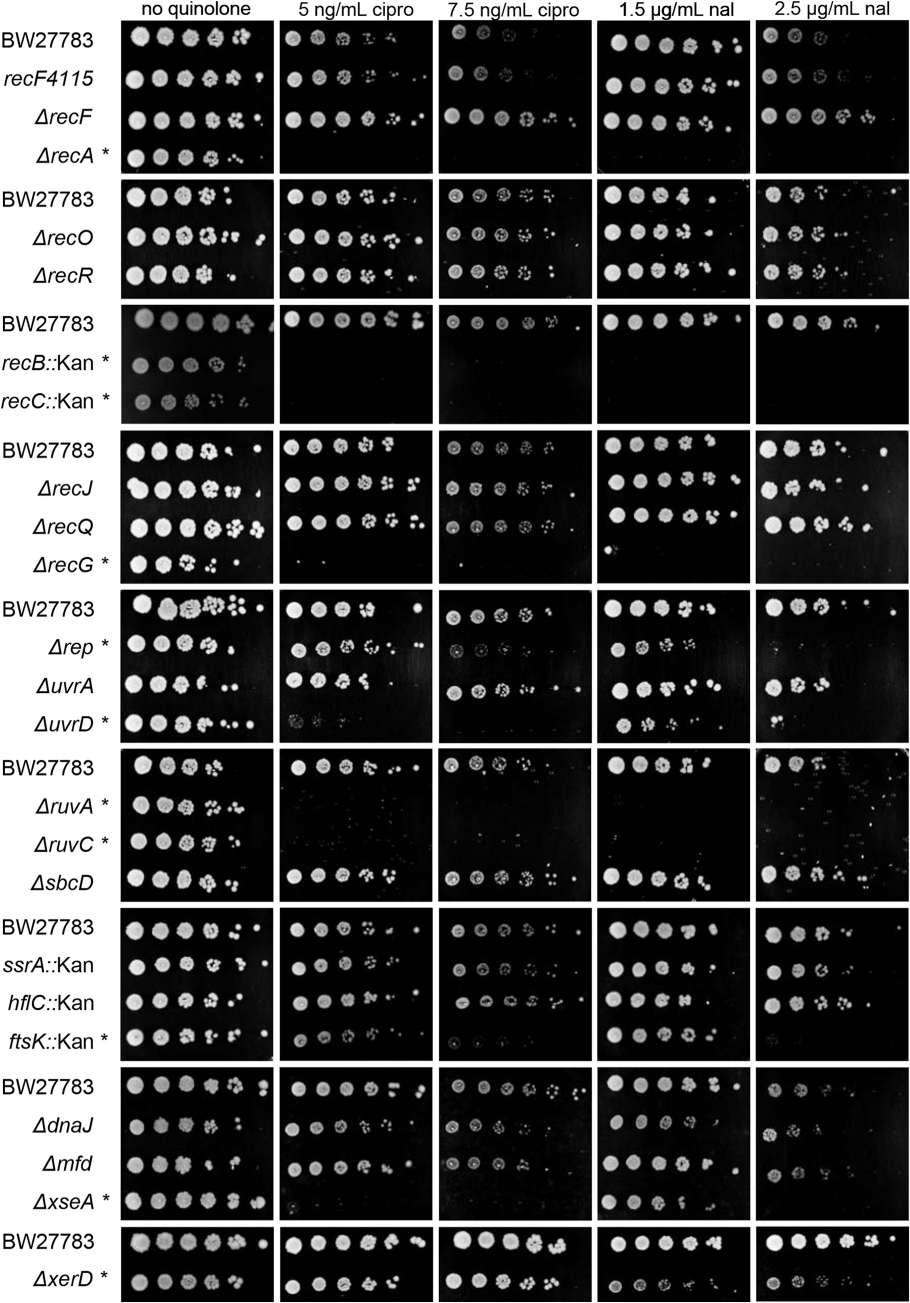
Sensitivity to quinolone antibiotics. Overnight cultures of BW27783 (wild-type) and the indicated BW27783 derivatives, were serial diluted five-fold and spotted onto LB plates containing either ciprofloxicin (5 or 7.5 ng/mL) or nalidixic acid (1.5 or 2.5 μg/mL) and incubated at 37° overnight. Those mutants deemed to be hypersensitive are indicated with an asterisk.

Strains with mutations in *recA*, *recB*, *recC*, *recG*, *uvrD*, *ruvA*, *ruvC*, *xseA*, *ftsK*, *xerD*, and *rep* were found to be hypersensitive to exposure to quinolone antibiotics (Fig 4; data summarized in Table 2). Knockouts in most of these genes have previously been shown to cause quinolone hypersensitivity (see citations above), but novel hypersensitive mutants emerged: *rep*, *ftsK* and *xerD*. As discussed above in the context of TBCs, hypersensitivity of both the *ftsK* and *xerD* mutants apparently reflects the importance of resolving chromosome dimers generated from homologous recombination between sister chromosomes, and this pathway is presumably critical following repair of quinolone-induced DNA breaks. The role of Rep in survival after quinolone treatment is intriguing, and could conceivably reflect either a direct role in favoring the dissociation of drug-stabilized gyrase cleavage complexes or a downstream role in replication restart after quinolone-induced fork blockage (or breakage).

**Table 2:** Summary of growth in the presence of quinolones.

Mutant	none	5 ng/mL cipro^a^	7.5 ng/mL cipro^a^	1.5 μg/mL nal^b^	2.5 μg/mL nal^b^
*recF4115*	+1	+1	+1	0	+2
*ΔrecF*	0	+1 ↑	+2 ↑	-1	+2 ↑
*ΔrecA* *	0	-6 ↓	-6 ↓	-5 ↓	-3 ↓
*ΔrecO*	+1	0	0	0	0
*ΔrecR*	0	+1	0	+1	0
*recB*::*Kan* *	-1 ↓	-6 ↓	-5 ↓	-5 ↓	-4 ↓
*recC*::*Kan* *	-1 ↓	-6 ↓	-5 ↓	-5 ↓	-4 ↓
*ΔrecJ*	0	+1	0	+1	-1
*ΔrecQ*	+1	+1	0	0	+1
*ΔrecG* *	-1	-5 ↓	-5 ↓	-4 ↓	-4 ↓
*Δrep* *	0	+2 ↓	-1 ↓	-1 ↓	-2 ↓
*ΔuvrA*	0	0	-1	0	0
*ΔuvrD* *	0	-1 ↓	-5 ↓	-1 ↓	-2 ↓
*ΔruvA* *	+1	-5 ↓	-4 ↓	-5 ↓	-3 ↓
*ΔruvC* *	+1	-5 ↓	-4 ↓	-5 ↓	-3 ↓
*ΔsbcD*	+1	0	+1	0	+2
*ssrA*::*Kan*	0	0	0	-1	0
*hflC*::*Kan*	0	0	0	-1	0
*ftsK*:*Kan* *	0	-1 ↓	-1 ↓	0	-3 ↓
*ΔdnaJ*	-1	-1	-1	0	-1
*Δmfd*	-1	-1	-2	0	0
*ΔxseA* *	0	-4 ↓	-6 ↓	0	-4 ↓
*ΔxerD* *	0	-1	0	0 ↓	-1 ↓

For ease of comparison, the results of dilution plating (from Fig 4) were categorized in comparison to the wild-type dilution series on the same plate. The number in each entry indicates which dilution tube showed growth (more than one colony) relative to the wild-type series (+1 indicates that the mutant grew in the next more dilute spot than the wild type while -1 indicates that the mutant grew in the next more concentrated spot; note that each dilution step was five-fold, and so a reading of -3 would correspond to a dilution factor differential of 125). The downward arrow next to the number indicates that the colonies that did grow were noticeably smaller than the corresponding wild-type colonies on the same plate (or that no colonies grew in the corresponding mutant spot); the upward arrow for the *ΔrecF* spots indicated that the colonies grew larger than the corresponding wild-type colonies. Both negative numbers and downward arrows provide evidence for hypersensitivity.

* Mutants that we designated as hypersensitive are indicated by an asterisk.

^a^ Ciprofloxacin, cipro

^b^ Nalidixic acid, nal

We obtained some useful negative data, namely that mutations in *recF*, *recO*, *recR*, *recJ*, *recQ*, *uvrA*, *sbcD*, *dinG*, *dnaJ*, *helD*, *hflC*, *mfd*, *polB* and *ssrA* cause little or no hypersensitivity to quinolones (Fig 4; Table 2; S1 Fig). The quinolone analyses also provide good internal controls for the data on sensitivity to TBCs formed by M.EcoRII-C186A, in the sense that every mutant that had been previously tested for quinolone sensitivity behaved as expected. However, it should be noted that a strain lacking SbcCD was previously shown to be modestly hypersensitive to killing by nalidixic acid (but not ciprofloxacin), while the minimal inhibitory concentration was unaffected [95]. Also, in a spot test comparable to our approach, an SbcCD mutant was not hypersensitive to ciprofloxacin [67].

We noted an interesting anomaly that should be considered whenever using the *recF* mutant from the Keio collection [62]. The deletion strain was noticeably resistant to quinolones, in contrast to *recR* or *recO* mutants, which behaved like wild type (Fig 4; Table 2). Inspection of the sequence of the deletion revealed that part of the promoter for the downstream *gyrB* gene is deleted, presumably leading to lower levels of DNA gyrase (and hence resistance to the poisoning effect of quinolones). To gauge the effect of a simple loss of RecF function, we moved the null *recF4115* point mutant [64] into this background, and found that the mutant displays wild-type sensitivity to quinolones. This anomaly concerning the Keio *recF* mutant presumably led to an incorrect conclusion about the role of RecF in persistence with quinolone treatment in a recent study [96]. In the above study of TBCs induced by M.EcoRII-C186A, the Keio deletion and *recF4115* displayed similar hypersensitivities, confirming the importance of RecF function in that assay.

## Further Discussion

Based on this study of TBCs induced by M.EcoRII-C186A and past studies of aza-C-induced DPCs induced by wild-type M.EcoRII, we now have a direct comparison of cellular functions that protect against the same tightly bound protein in either non-covalent or covalent form (summarized in Table 3). Several gene products are needed for protection against both kinds of complexes, notably RecA, RecBC, RecG, RuvABC, UvrD, FtsK, XerD, and SsrA (tmRNA).

**Table 3:** Sensitivity to M.EcoRII-C186A induced TBCs, quinolones, and aza-C induced DPCs.

	TBCs (Fig 3)	Quinolones (Fig 4)	M.EcoRII DPCs (aza-C-induced)
***dinG***	wild-type	wild-type	hypersensitive [52]
***dnaJ***	hypersensitive	wild-type	hypersensitive [51]
***ftsK***	hypersensitive	hypersensitive	hypersensitive [52]
***helD***	wild-type	wild-type	not reported
***hflC***	wild-type	wild-type	hypersensitive [51]
***mfd***	wild-type	wild-type	wild-type [49,51]
***polB***	wild-type	wild-type	wild-type [49]
***recA***	hypersensitive	hypersensitive	hypersensitive [46,47,48,49,52]
***recBC***	hypersensitive	hypersensitive	hypersensitive [49,52]
***recFOR***	hypersensitive	wild-type	wild-type [48,52]
***recG***	hypersensitive	hypersensitive	hypersensitive [49,52]
***recJ***	wild-type	wild-type	wild-type [49]
***recQ***	wild-type	wild-type	wild-type [49]
***rep***	hypersensitive	hypersensitive	wild-type [52]
***ruvABC***	hypersensitive	hypersensitive	hypersensitive [49,52]
***sbcCD***	wild-type	wild-type	wild-type [52]
***ssrA***	hypersensitive	wild-type	hypersensitive [51]
***uvrA***	wild-type	wild-type	wild-type [46,47,48,49,51]
***uvrD***	hypersensitive	hypersensitive	hypersensitive [49,51]
***xerCD***	hypersensitive	hypersensitive	hypersensitive [52]
***xseA***	wild-type	hypersensitive	not reported

This table summarizes the sensitivity of various *E*. *coli* mutants to TBCs induced by M.EcoRII (Fig 3; this study), topoisomerase cleavage complexes (Fig 4; this study; also see citations in the text to past studies of quinolone-sensitive mutants), and DPCs induced by wild-type M.EcoRII proteins with aza-C treatment (indicated citations).

The first group of functions (RecA, RecBC, RecG and RuvABC) could play key roles in processing blocked replication forks, or perhaps more likely, downstream DNA breaks resulting from blocked forks, given that both kinds of M.EcoRII complex block replication forks [8]; (Fig 2). Breaks could be generated by nuclease cleavage of the stalled fork and/or by collision of a subsequent replication fork with the stalled fork [30]. The detection of Y forms and X structures with M.EcoRII DPCs but not TBCs implies that such fork breakage events are much more common with forks stalled at covalently bound proteins, which would not be surprising given the presence of the covalent protein-DNA bond in the DPC. The hypersensitivity of FtsK and XerD knockouts to both DPCs and TBCs implies a key role of chromosome dimer resolution, strongly suggesting that the homologous recombination functions trigger sister chromosome recombination in response to these DNA lesions.

Involvement of the tmRNA pathway indicates that both kinds of complex also block the coupled transcription-translation machinery (see [51]). The importance of UvrD to survival with both kinds of protein complexes might also relate to transcription blockage, since UvrD (and its homolog PcrA) interacts specifically with RNA polymerase [22]. In addition, UvrD was recently shown to push RNA polymerase backwards at blocking lesions and template-bound proteins, promoting excision repair of the blocking lesions [23]. Perhaps UvrD also licenses removal of a template-bound protein encountered by RNA polymerase (although the helicase is on the wrong strand for this activity when it is pushing RNA polymerase backwards). Survival and growth might be promoted by releasing stalled RNA polymerase at sites remote from replication forks and/or by preventing collisions of the replication fork with stalled RNA polymerase. Another possible role for UvrD, not mutually exclusive, is that the helicase prevents untoward recombination events after fork blockage by removing excess RecA protein from the fork region [32,77,97]

Very notable differences were observed between the responses to the two kinds of M.EcoRII complexes (Table 3). Of particular interest, Rep helicase and the RecFOR pathway were found to be important in the response to TBCs induced by M.EcoRII-C186A but not to DPCs induced by aza-C with the wild-type protein. Rep helicase specifically interacts with DnaB helicase at the fork to promote the dissociation of blocking proteins [14,24]. However, Rep translocates on single stranded DNA in the 3’ to 5’ direction [98], and so it needs to gain access to the leading strand template, where the replicative polymerase is normally engaged, in order to progress towards a blocking protein. It is not clear whether Rep is always associated with the replisome or needs to be recruited to the replisome when needed. Even if it is always associated with the replisome, it apparently needs to be activated, presumably by loading onto the leading strand template, to explain the prominence of fork blockage in vivo at M.EcoRII TBCs (Fig 2). The mechanism of activating Rep helicase at a blocked fork is currently unknown.

The function of RecA at stalled forks might provide a hint concerning this mechanism. As described in the Introduction, RecA was shown to play key roles at forks stalled by damage such as pyrimidine dimers. More recently, Indiani et al. showed that RecA at a stalled fork licenses the action of translesion polymerases including PolIV while inhibiting the action of PolIII, acting as a “master switch” or “traffic cop” in their words [99]. Furthermore, Tan et al. showed that the replication fork slows down in a RecA-dependent fashion during the SOS response, again pointing to an important role of RecA at replication forks with template damage [100]. While speculative, we suggest that perhaps RecA can also facilitate the loading of Rep onto the leading strand template, perhaps in part by its ability to inhibit or dislodge PolIII, in order to promote dissociation of tightly bound proteins that are blocking the fork. Interestingly, Rep has been shown to also interact with PolIV, with PolIV stimulating Rep helicase activity [101]. This raises the possibility of a more extensive multi-protein complex, centered on traffic cop RecA, which adeptly solves many different problems at the replication fork.

If RecA action at the fork licenses the action of Rep helicase, the involvement of RecFOR in the response to M.EcoRII TBCs can be explained by its well-known ability to load RecA onto single-stranded DNA. Indeed, Morimatsu et al. directly showed that RecFOR can load RecA onto lagging strand gaps adjacent to Okazaki fragments [102].

Why are RecFOR and Rep dispensable for growth with tandem arrays of repressor binding sites in the chromosome [13]? Repressor binding in the arrays is weaker and more rapidly reversible than M.EcoRII-C186A binding, and this may substantially reduce the need for the accessory helicase (and RecA loading). Indeed, each repressor-loaded binding site in an array was estimated to block only about 5–10% of replication forks in vitro, DNA replication was shown to progress well into an occupied array in vivo [12], and the amount of fork blockage was dependent on the length of the array (binding site copy number) [13]. Therefore, replication may advance “site-by-site” as repressors dissociate from the site nearest the fork, without need for the accessory helicase. Nonetheless, this pathway appears to be insufficient as the array of binding sites gets longer and longer, since arrays of 240 sites completely blocked DNA replication [103].

A direct comparison of sensitivity to M.EcoRII protein within DPCs versus TBCs also revealed a novel role for DinG helicase as well as the HflC chaperone in survival after DPC formation (Table 3). These two proteins apparently play some special role that is dictated by the existence of the covalent DNA-protein bond. One possibility is that they act directly on the DPC, perhaps with DinG playing a role in DPC recognition along the DNA and HflC acting on the covalently bound protein after recognition. Two recent studies revealed a pathway for DPC repair involving proteolysis of the covalently bound protein in eukaryotic cells [104,105], raising the exciting possibility that HflC acts directly on the covalently bound protein in *E*. *coli*. A different interpretation that might explain the role of DinG is that this helicase plays some important role in rescuing the more frequent fork breakage events that seem to occur with the DPC.

In closing, we would like to highlight some interesting new hypotheses that emerge from this research: (1) stalled fork processing by RecFOR and RecA promotes release of tightly bound (but non-covalent) blocking proteins, perhaps by a mechanism involving Rep activation by RecA; (2) a covalent bond between DNA and the blocking protein increases the likelihood of fork breakage; (3) TBC-blocked forks generate broken ends by a RuvABC-independent process (cleavage by another nuclease or rear-end collisions from a later replication fork); (4) DinG plays a special role in DPC repair or survival, conceivably relating to DPC recognition; (5) HflC plays a special role in DPC repair, perhaps acting on the covalently attached protein in a proteolysis pathway; (6) the tmRNA system is induced when RNA polymerase is blocked by TBCs; (7) the Keio *recF* mutant has decreased expression of GyrB, leading to moderate quinolone resistance.

## Supporting Information

S1 Fig Sensitivity to M.EcoRII-C186A and quinolone antibiotics. Overnight cultures of BW27783 (wild-type) and the indicated BW27783 derivatives, containing plasmid pBAD-MEcoRII-C186A, were serial diluted five-fold and spotted onto LB plates containing chloramphenicol with either glucose (0.01%) or arabinose (0.0003%, 0.001%, 0.003%, or 0.01%). The same dilutions were also spotted onto LB plates containing either ciprofloxicin (5 or 7.5 ng/mL) or nalidixic acid (1.5 or 2.5 μg/mL). All plates were incubated at 37° overnight.
(TIF)
