# Supplementary figures and images for: Functions that Protect Escherichia coli from Tightly Bound DNA-Protein Complexes Created by Mutant EcoRII Methyltransferase
Source: PLoS One. 2015 May 19;10(5):e0128092. doi: 10.1371/journal.pone.0128092 (PMC4437897; doi:10.1371/journal.pone.0128092)

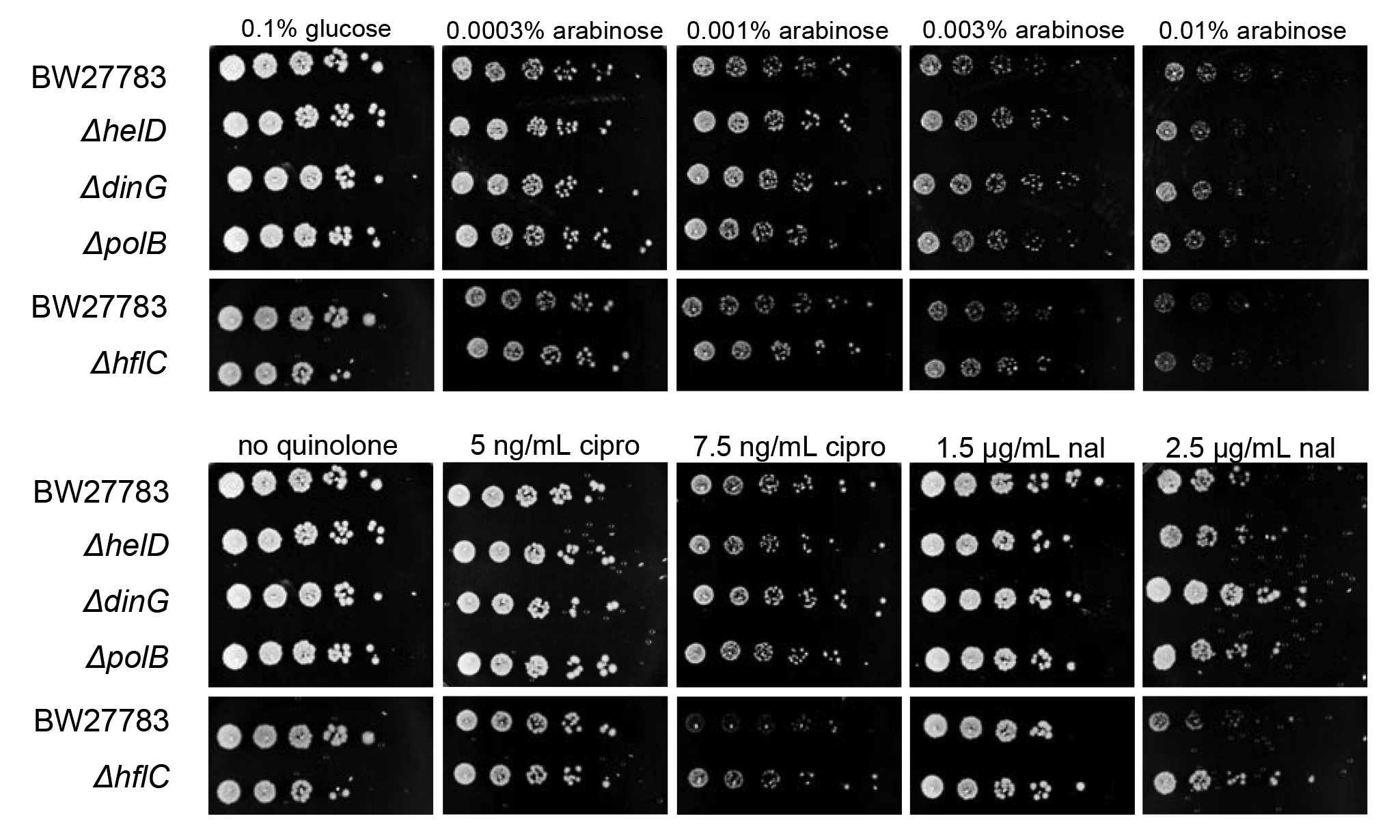

Supplement: S1 Fig — Overnight cultures of BW27783 (wild-type) and the indicated BW27783 derivatives, containing plasmid pBAD-MEcoRII-C186A, were serial diluted five-fold and spotted onto LB plates containing chloramphenicol with either glucose (0.01%) or arabinose (0.0003%, 0.001%, 0.003%, or 0.01%). The same dilutions were also spotted onto LB plates containing either ciprofloxicin (5 or 7.5 ng/mL) or nalidixic acid (1.5 or 2.5 μg/mL). All plates were incubated at 37° overnight. (TIF) [file pone.0128092.s001.tif]
